# A novel germline *ARMC5* mutation in a patient with bilateral macronodular adrenal hyperplasia: a case report

**DOI:** 10.1186/s12881-018-0564-2

**Published:** 2018-03-27

**Authors:** Qiuli Liu, Dali Tong, Jing Xu, Xingxia Yang, Yuting Yi, Dianzheng Zhang, Luofu Wang, Jun Zhang, Yao Zhang, Yaoming Li, Lianpeng Chang, Rongrong Chen, Yanfang Guan, Xin Yi, Jun Jiang

**Affiliations:** 1 Department of Urology, Institute of Surgery Research, Daping Hospital, Third Military Medical University, 10#, Changjiang Zhilu, Yuzhong District, Chongqing, 400042 People’s Republic of China; 2 Geneplus-Beijing Institute, Beijing, 102206 People’s Republic of China; 3 Department of Bio-Medical Sciences, Philadelphia College of Osteopathic Medicine, 4170 City Avenue, Philadelphia, PA 19131 USA

**Keywords:** Bilateral macronodular adrenal hyperplasia, Cushing’s syndrome, *ARMC5*

## Abstract

**Background:**

Bilateral macronodular adrenal hyperplasia (BMAH) is a rare cause of Cushing’s syndrome (CS). BMAH is predominantly believed to be caused by two mutations, a germline and somatic one, respectively, as described in the two-hit hypothesis. In many familial cases of BMAH, mutations in armadillo repeat containing 5 (*ARMC5*), a putative tumor suppressor gene, are thought to induce the disorder. The objective of this study was to report a case in which the patient presented with BMAH induced by a novel heterozygous germline *ARMC5* mutation (c. 517C > T, p. Arg173*) alone rather than a two-hit mutation.

**Case presentation:**

A 51-year-old woman was identified with masses in the bilateral adrenals. Serum cortisol levels were increased significantly both in the morning (08:00 AM) and late at night (24:00 AM), while plasma adrenocorticotropic hormone was normal. The patient underwent a left adrenalectomy and histopathology substantiated the BMAH diagnosis. WES of the germline DNA discovered a novel heterozygous germline *ARMC5* mutation (c. 517C > T, p. Arg173*) and in silico analysis predicted that the mutation significantly impaired protein function, resulting in inactivated ARMC5. Subsequently, WES of the tumor specimen identified 79 somatic single nucleotide polymorphisms (SNPs)/insertion-deletion (indel) mutations, including 32 missense/nonsense/splice/stop-loss mutations. None of these mutations were CS-related.

**Conclusions:**

A novel germline *ARMC5* mutation (c. 517C > T, p. Arg173*) was identified that induced BMAH alone without a second mutation. ARMC5 sequencing may improve the identification of clinical forms of BMAH and allow earlier diagnosis of this disease.

**Electronic supplementary material:**

The online version of this article (10.1186/s12881-018-0564-2) contains supplementary material, which is available to authorized users.

## Background

Bilateral macronodular adrenal hyperplasia (BMAH) is a rare type of Cushing’s syndrome (CS) that accounts for less than 1% of all endogenous CS cases [1]. BMAH is characterized by bilateral enlargement of the adrenal glands, with adrenocortical nodule diameters larger than 10 mm and frequently closer to 30 or 40 mm. Patients with BMAH also present with abnormal cortisol production and reduced expression of steroidogenic enzymes and Melanocortin type 2 receptor (MC2R) [2]. Usually, the disease progresses slowly, and patients are diagnosed between 40 and 60 years old only after they present with CS and suppressed circulating corticotropin levels [3]. One potential reason for the slow disease progression is that increased overall cortisol production only occurs when the patients have developed large adrenal nodules [3]. Indeed, most BMAH patients are identified while investigating for an incidentally discovered bilateral adrenal lesion. Importantly, the bilateral appearance of the nodules raised the hypothesis of an underlying germline genetic predisposition. Although most cases appear sporadic, familial cases are well documented, indicating a potential major genetic component of the disease [4].

Many familial cases of BMAH are associated with mutations in *ARMC5*, a putative tumor suppressor gene, which are thought to underlie this form of the disorder [3]. About 45–55% of BMAH cases are thought to be associated with the variations in ARMC5 [3, 5]. These mutations inactivate *ARMC5* and slowly induce dedifferentiation of adrenocortical cells and growth of bilateral masses. *ARMC5*-mutant patients also present with a more severe disease than wild-type patients. Specifically, in the mutated group, the age of diagnosis is lower, while the prevalence of clinical CS and hypertension, the total adrenal weights, and the number of nodules are all higher [4].

Inactivation of ARMC5 in BMAH follows the “two-hit” model of a tumor suppressor gene responsible for a hereditary neoplasia syndrome. BMAH patients have a single germline mutation in ARMC5 leading to the expression of an inactive mutant protein, acting as the first ‘hit’; however, the other allele could encode for the protein. The BMAH would happen only if the other allele suffered another subsequent somatic mutation which resulted in the absence of the protein and causing the development of nodules and overproduction of cortisol [6]. Moreover, tumors caused by mutations in *ARMC5* are likely to be polyclonal, because both alleles of *ARMC5* carry mutations at both the somatic and germline levels. Therefore, different nodules on the same adrenal gland may carry different variations of the *ARMC5* gene [7]. In this study, we present a patient with BMAH induced by a novel heterozygous germline *ARMC5* mutation (c. 517C > T, p. Arg173*) alone rather than the previously hypothesized two-hit somatic mutation.

## Case presentation

A 51-year-old woman was admitted to our department for 2 months to identify masses in the bilateral adrenals. Physical examination revealed typical Cushingoid features, including a full moon face, central obesity, and purple striae of the bilateral axillary and lower abdomen.

Upon admission, the hormonal work-up revealed hypercortisolism both in the morning and late at night. Specifically, 8:00 morning serum cortisol levels were 604 nmol/L (normal range: 50-280 nmol/L) and 24:00 levels were 443 nmol/L (normal range: 10-120 nmol/L) (Table 1). Plasma ACTH levels were normal at both time points, at 11.72 pg/mL (normal range: 5.08-32.8 pg/mL) and 12.27 pg/mL (normal range: 5-15 pg/mL), for morning and night levels, respectively. The results of low- and high-dose dexamethasone suppression tests were negative, indicating the latter mentioned adrenal masses were corticotropin independent. Levels of other hormone were also abnormal, including Thyroxine (T4): 41.83 nmol/L (normal range: 78.38-157.4 nmol/L) and free T4: 5.16 pmol/L (normal range: 7.84-20.1 pmol/L), while estradiol, testosterone, FSH (follicle-stimulating hormone), LH (luteinizing hormone), growth hormone, thyrotropin (TSH), and prolactin (PRL) levels were all normal.

**Table 1:** Patient hormone expression profile

Hormone (reference range)	On first admission	After left adrenalectomy	On second admission
Cortisol 8, ng/mL (50–280 ng/mL)	604	244	217
Cortisol 16, ng/mL (20–140 ng/mL)	361	294	252
Cortisol 24, ng/mL (10–120 ng/mL)	443	273	281
ACTH 8, pg/mL (5.08–32.8 pg/mL)	11.72	7.21	7.06
ACTH 16, pg/mL (10.7–30.5 pg/mL)	9.25	9.17	10.44
ACTH 24, pg/mL (5–15 pg/mL)	12.27	9.25	5.25
Vanillylmandelic acid, μmol/24 h (9.6-50.0 μmol/24 h urine)	23.40	–	29.80
17-hydroxycorticosteroid, μmol/24 h (5.5-33.2 μmol/24 h urine)	14.30	–	15.80
17-ketosteroid, μmol/24 h (13.9-76.3 μmol/24 h urine)	43.90	–	52.70
Orthostatic renin activity, ng/mL/h (0.33-5.15 ng/mL/h)	5.96	–	9.19
Clinostatic renin activity, ng/mL/h (0.07-1.51 ng/mL/h)	2.25	–	15.88
Thyroxine, T4, nmol/L (78.38-157.4 nmol/L)	41.83	–	–
Free thyroxine, nmol/L (7.86-20.1 nmol/L)	0.1	–	–

Abbreviations: *ACTH* adrenocorticotropic hormone

**Fig. 1:**
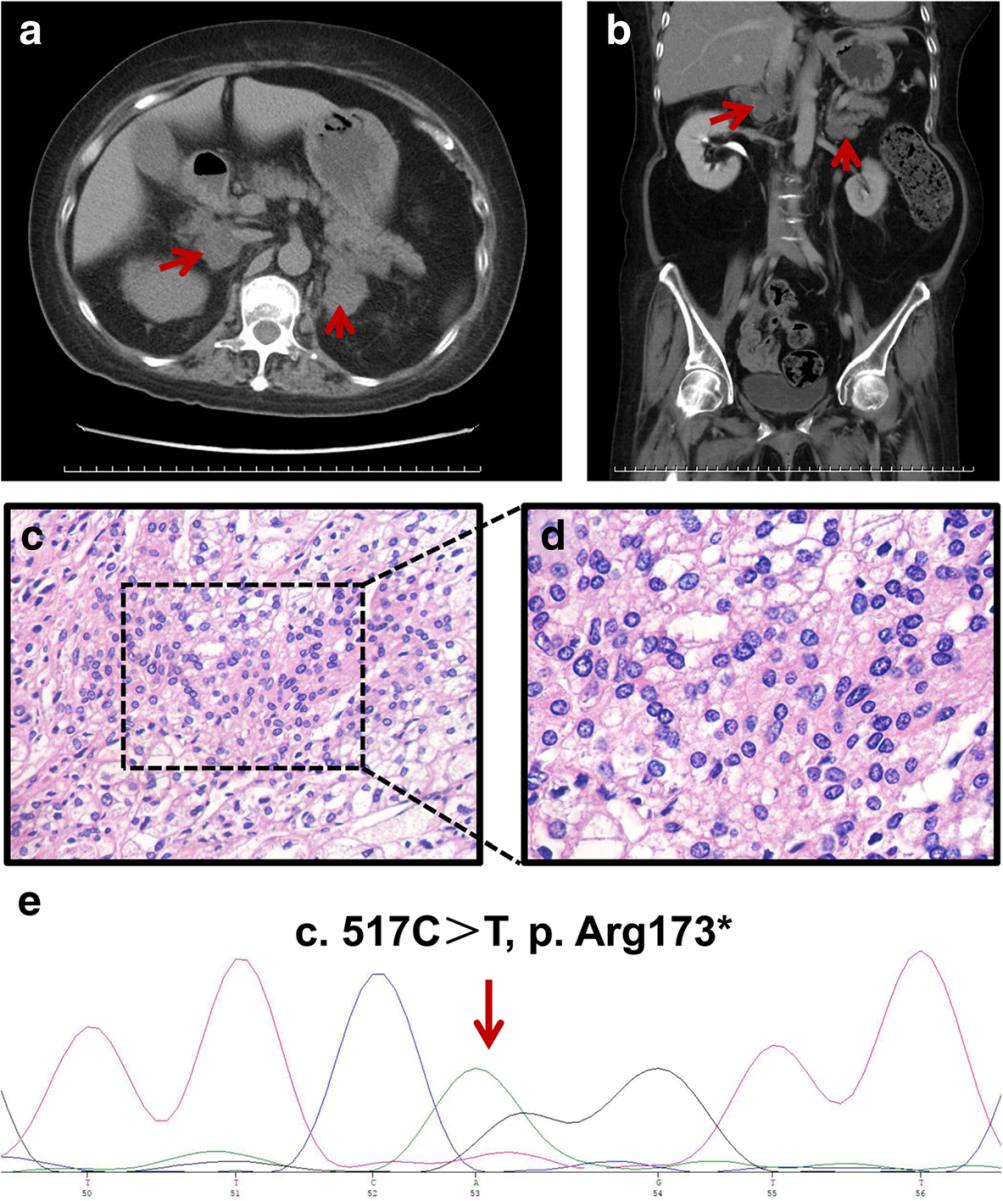
Results of imaging, histopathology, and DNA sequencing. **a** and **b** Adrenal computed tomography scan revealed bilateral adrenal masses (indicated by red arrows). **c** and **d** Hematoxylin and eosin staining of the adrenal tissue revealed nodular hyperplasia. **e**. Sequencing of DNA extracted from peripheral leukocytes identified a novel heterozygous germline *ARMC5* mutation, c. 517C > T, p. Arg173*. (indicated by red arrows)

Adrenal computed tomography (CT) revealed bilateral adrenal masses (left: 3.2*2.4 cm and right: 2.2*2.1 cm) (Fig. 1a and b), while chest CT revealed an old right rib fracture. Pituitary magnetic resonance imaging (MRI) was normal, whereas vertebral MRI revealed a compression fracture of the thoracolumbar spine (T7 and L3).

On January 19th, 2016, the patient underwent a left adrenalectomy using the retroperitoneal laparoscopic procedure. Surgically removed adrenal glands demonstrated nodular hyperplasia without hemorrhage or infarction, and histopathology substantiated the BMAH diagnosis (Fig. 1c and d).

Precise isolation of the tumor specimen during the sample collection was conducted with the help of the H&E staining. Using WES (whole-exome sequencing) of the germline and tumor DNA, we first identified a novel heterozygous germline *ARMC5* mutation (c. 517C > T, p. Arg173*, chr16: 31473284) that resulted in a premature translational-termination codon. This nonsense mutation would impair the function of ARMC5. The *ARMC5* variants identified by WES were validated by Sanger sequencing of the germline DNA (Fig. 1e). We then performed segregation analysis of the variants. All coding exons of *ARMC5* in the patient were sequenced, and then this sequence was analyzed using Mutation Surveyor version 2.51 (SoftGenetics LLC) with comparison to the reference sequence in GenBank (http://www.ncbi.nlm.nih.gov/genbank/) Subsequently, we conducted WES of the tumor specimen compared to matched normal DNA and identified 79 somatic single nucleotide polymorphisms (SNPs)/insertion-deletion (indel) mutations, including 32 missense/nonsense/splice/stop-loss mutations (Additional file 1: Table S1). Among these, In silico analysis predicted no known CS-related mutations. The CARE guidelines were followed when reporting this case.

## Discussion and conclusion

In this study, we reported a rare case of BMAH in which the patient presented with the typical bilateral adrenal macronodular hyperplasia, BMAH-associated CS, and hypertension. In this unique case of BMAH, the sequencing results identified a novel heterozygous germline *ARMC5* mutation (c. 517C > T, p. Arg173*).

As these adrenal nodules inefficiently produce cortisol, a clinical syndrome only becomes apparent with large nodules. Previous studies predicted that this form of BMAH becomes clinically apparent only with a second somatic mutation in addition to *ARMC5*, as described in Knudson’s two-hit model [3]. In these cases, *ARMC5* would bear a first germline mutation and a second somatic mutation within the adrenals, resulting in a loss of protein function and subsequent tumor formation [8]. However, in this case, BMAH development did not seem to require a second mutation. The patient presented with only a heterozygous germline *ARMC5* mutation (c. 517C > T, p. Arg173*), and no mutations of the other allele or of CS-related genes in the bilateral adrenal tissues. This single germline *ARMC5* mutation is not entirely surprising, as Zilbermint et al. previously reported a heterozygous germline *ARMC5* mutation with no additional somatic inactivating events in *ARMC5* exons, such as loss of heterozygosity [7]. This cast doubt on the pathogenic role of heterozygous *ARMC5* mutations in primary aldosteronism. Our results suggest that *ARMC5*-induced BMAH does not require additional somatic mutations, which was consistent with the haploinsufficient model [9]. The combination of high-frequency LOH and rare point mutations has led to the suggestion that *ARMC5* might demonstrate haploinsufficiency (absent or reduced function due to the loss or inactivation of a single allele), just as *CDKN1B* described by A. J. W. Paige [9]. However, this needs to be further substantiated. Of course, similar to Correa et al. [10], we cannot not exclude the possibility that other larger genomic losses or the changes of the methylation, as well as structural alterations including inversions and copy number changes might exist that were not detected by the methods used in this study.

BMAH is a heterogeneous disease with varying degrees of hypercortisolism (from subclinical to overt CS), radiological aspects (from massive nodular hyperplasia to macronodular adrenal hyperplasia), and illegitimate receptors [11]. The presence or absence of *ARMC5* mutations may be associated with these diverse phenotypes. Previous studies have shown that ARMC5 defects are associated with a more severe disease, such as higher cortisol levels, larger adrenal glands and higher numbers of nodules [3, 5]. In addition, ARMC5-mutated patients have hypertension more often than WT (wild type) patients [12]. However, one important consideration is that even if there is a connection between BMAH and *ARMC5* mutations, the resulting phenotypes would vary with the nature of the mutations. For example, Elbelt el al. [13] reported that somatic (p.R502fs) and germline frame shift *ARMC5* mutations (c.323_324insC, p.A110fs*9) are associated with intracranial meningioma, while other family members lacking the somatic mutation did not develop the disease. This indicates that specific *ARMC5* mutations are associated with unique disease phenotypes. In the current study, the heterozygous germline *ARMC5* mutation (c. 517C > T, p. Arg173*) alone was potentially associated with younger age at diagnosis, BMAH, CS, and hypertension.

In conclusion, we have identified a novel germline *ARMC5* mutation (c. 517C > T, p. Arg173*) in a sporadic case of BMAH characterized with bilateral adrenal macronodular hyperplasia, BMAH-associated CS, and hypertension. Identification of this genotype-phenotype relationship increases our knowledge regarding the development of BMAH and the connection to ARMC5. Determining patient *ARMC5* status might enhance BMAH diagnosis, and screening family members for *ARMC5* mutations may enable clinicians to identify BMAH earlier and prevent patient morbidity and mortality.

## Additional file

Additional file 1 **: Table S1.** Identified 79 somatic single nucleotide polymorphisms (SNPs)/insertion-deletion (indel) mutations. Whole-exome sequencing of the tumor specimen compared to matched normal DNA was conducted and 79 somatic single nucleotide polymorphisms (SNPs)/insertion-deletion (indel) mutations were identified, including 32 missense/nonsense/splice/stop-loss mutations (XLSX 24 kb)

## Abbreviations

ACTH: Adrenocorticotropic hormone
*ARMC5*: Armadillo repeat containing 5
BMAH: Bilateral macronodular adrenal hyperplasia
CS: Cushing’s syndrome
CT: Computed tomography
FSH: Follicle-stimulating hormone
IRB: Institutional review board
LH: Luteinizing hormone
MC2R: Melanocortin type 2 receptor
MRI: Magnetic resonance imaging
PRL: Prolactin
PROG: Progesterone
SNPs: Single nucleotide polymorphisms
T4: Thyroxine
TESTO: Testosterone
WES: Whole-exome sequencing
